# Case report: The case report of ofatumumab, a fully human anti-CD20 monoclonal antibody, in the treatment of KLHL11 encephalitis

**DOI:** 10.3389/fimmu.2024.1456840

**Published:** 2024-11-07

**Authors:** Min Deng, Fei Zeng, Zhaohong Kong, Tao Li

**Affiliations:** Department of Neurology, Renmin Hospital of Wuhan University, Wuhan, China

**Keywords:** KLHL11, encephalitis, ofatumumab, CD20, B cell

## Abstract

Anti-kelchlike protein 11 (KLHL11) encephalitis was first reported in 2019. This disease is very rare. The prevalence is higher in men than in women. Few female cases have been described. The most common clinical manifestations of this disease are syndromes affecting the brainstem and cerebellum. There are few reports on epilepsy and hallucinations as main clinical manifestations of KLHL11 encephalitis. We report a case of KLHL11 encephalitis in a female with epilepsy and hallucinations as the primary symptom. Her EEG showed a large number of epileptiform waves could be seen in the bilateral temporal and sphenoid areas during the waking and sleeping stages. Her head MRI was normal. No tumors were found using PET/CT scan and tumor marker measurements. Her symptoms recurred and worsened soon after treatment with immunoglobulin and methylprednisolone. Fortunately, epilepsy and hallucinations were effectively controlled after six times of subcutaneous injection of ofatumumab. Ofatumumab is a CD20 monoclonal antibody that induces B cell depletion. Current studies show that KLHL11 encephalitis is mediated by T cell immunity. However, in this case, satisfactory clinical effects were observed using CD20 monoclonal antibodies to treat KLHL11 encephalitis. This is the first report of induced B cell depletion in the treatment of KLHL11 encephalitis. This may provide a potential treatment option for KLHL11 encephalitis.

## Introduction

Anti-kelchlike protein 11 (KLHL11) antibodies were first discovered in 2019 by Deutscher and Mandel-Brehm et al (1). This antibody is directed against the intracellular KLHL11 antigen, which is commonly associated with malignant tumor (2). KLHL11 antibody encephalitis, also known as KLHL11 antibody-associated paraneoplastic syndrome (PNS), is a very rare disease. Anti-KLHL11 antibodies can be identified by serum and cerebrospinal fluid antibody testing. Due to its intracellular localization, the primary pathogenic mechanism of KLHL11-PNS is thought to be T cell mediated (3). Clinical manifestations include cerebellar syndrome with brainstem symptoms, in addition to which symptoms may be associated with hearing loss, diplopia, vertigo, and ataxia. Studies have reported that KLHL11 antibodies are closely associated with testicular germ cell tumors (1), and few female cases have been described so far. Treatment of the disease is less effective. Most patients with KLHL11 encephalitis respond poorly to immunotherapy and tumor treatments, seriously affecting the effectiveness of treatment (3). In this case, the patient was female and had a poor response to conventional immunoglobulin therapy. After a short-term relapse, we treated this patient with ofatumumab. Ofatumumab is a fully human anti-CD20 monoclonal antibody that was approved for marketing in 2009 for the treatment of chronic lymphocytic leukemia (4). In addition, this monoclonal antibody has also been used to treat rheumatoid arthritis (5) and multiple sclerosis (6) in recent years. However, the efficacy of ofatumumab in KLHL11-positive encephalitis has not been reported. The case we report is a patient with KLHL11-positive encephalitis who relapsed shortly after immunoglobulin therapy. While she has received six doses of ofatumumab, she has had no recurrences or infections so far.

## Case presentation

A 66-year-old female patient began to experience narcolepsy in April 2022. Each episode of drowsiness lasted 10-30 minutes and occurred once every 2-3 days. She often had hallucinations and used to feel like there were strangers walking around her home. She had many dreams at night and shouted in her sleep. She went to a psychiatry department and was diagnosed with anxiety disorder and sleep disorder. She received oral treatment with sertraline and tandospirenone citrate tablets. She began to experience episodic falls accompanied by convulsions in June 2023. This symptom lasted for about 1 minute and resolved on her own, and occured 5-10 times a day. She often had hallucinations. The symptoms of episodic falls gradually worsened, and he was hospitalized on June 20, 2023. She suffered a minor concussion due to trauma in 1998 and suffered from cystitis in 2010. She denied poisoning and family illness.

**Figure 1:**
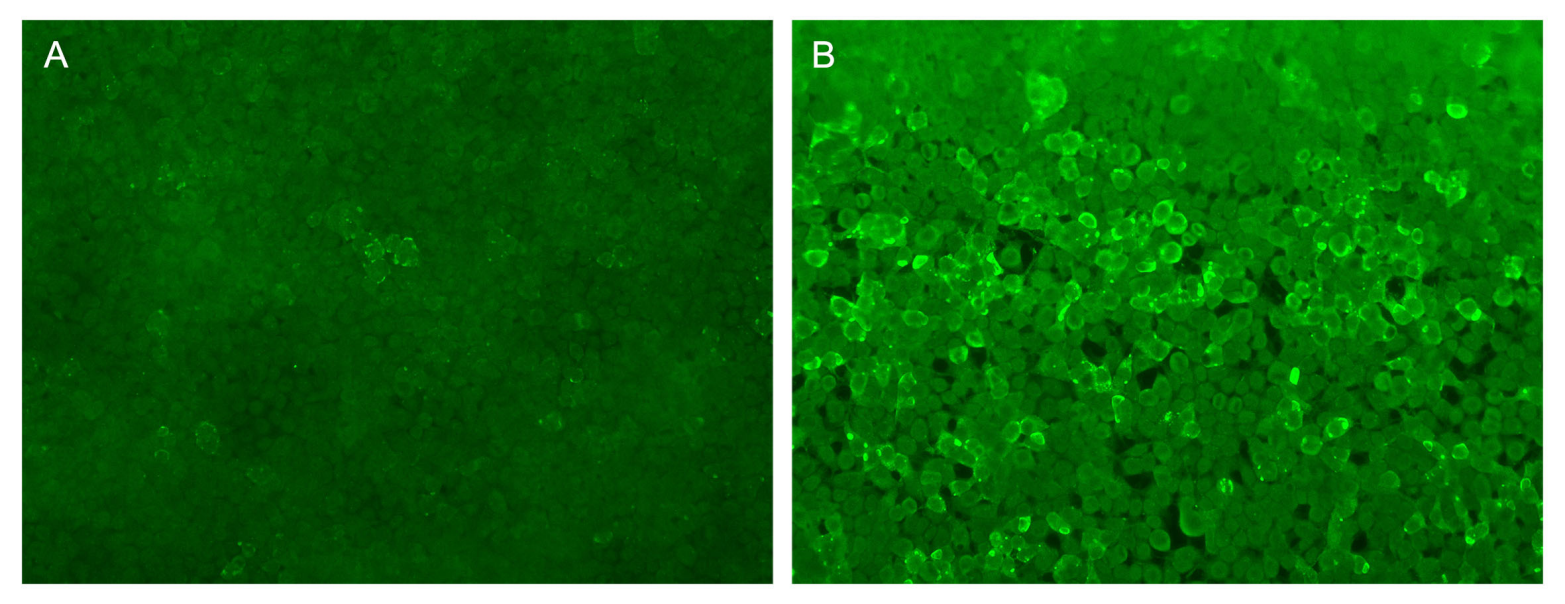
The fluorescence images of KLHL-11 IgG positive in serum and cerebrospinal fluid. **(A)** Fluorescent antibody staining for expression of KLHL-11 antibody in the first CSF of the patient. **(B)** Fluorescent antibody staining for expression of KLHL-11 antibody in the first blood of the patient.

The electroencephalogram showed that a large number of epileptiform waves could be seen in the bilateral temporal and sphenoid areas during the waking and sleeping stages. Analysis of cerebrospinal fluid showed normal intracranial pressure (165 mm H2O), normal leukocytes (1×10^6^), normal protein (0.28g/l) and normal glucose (2.75mmol/l). Lupus anticoagulant factor LA1 28.6sec (reference value 31-44sec), lupus anticoagulant factor LA2 29.6sec (reference value 30-38sec), antinuclear antibody 1:320 (reference value <=1:100), anti-SSA antibody 6.5 AI (reference value <1 AI), anti-SSA60 antibody 6.5AI (reference value <1 AI). Neuron-specific enolase 23.9ng/ml (reference value 0-16.3ng/ml). The remaining tumor markers were negative (including human epididymal protein 4, abnormal prothrombin, alpha-fetoprotein, glycan antigen 125, glycan antigen 153, glycan antigen 199, glycan antigen 50, glycan antigen 72-4, carcinoembryonic antigen, squamous cell-associated antigen, cytokeratin fragment 19). The transfection cytology methods were used to detect autoimmune encephalitis antibodies (NMDAR, AMPAR1, AMPAR2, LGI1, CASPR2, GABABR, DPPX, IgLON5, GlyRα1, GABAARα1, GABAARβ3, mGluR1, mGluR5, D2R, Neurexin-3α, GAD65, KLHL11, gAChR, AQP4, MOG, GFAP) in cerebrospinal fluid and serum. KLHL11 IgG was detected positive in her serum (1:100, **Figure 1B**) and cerebrospinal fluid (1:1, **Figure 1A**).

**Figure 2:**
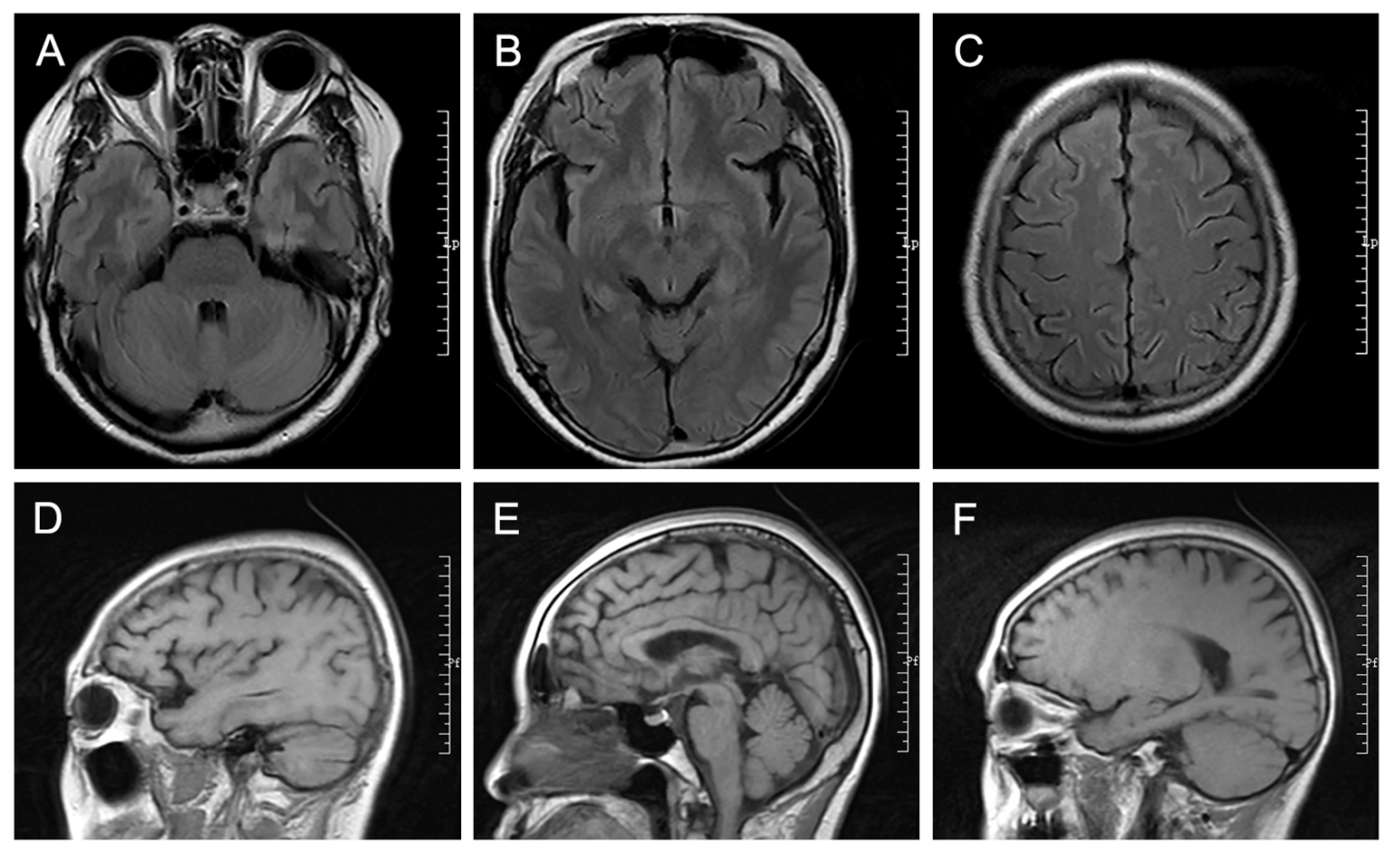
The radiology images. **(A-C)** The cross-sectional T2 FLAIR MRI. **(D-F)** The sagittal T1 FLAIR MRI.

Other test indicators showed no abnormalities, including blood cell count, liver function, renal function, blood lipids, blood glucose, thyroid function, muscle enzymes, antineutrophil cytoplasmic antibodies, Aβ amyloid 1-42, human phosphorylated tau-181. and coagulation function. MRI scans of the head were normal (**Figure 2**). The electrocardiogram was normal. Color ultrasound of the heart was normal. Ultrasound of the vaginal uterine appendages was normal. The PET/CT examination of whole-body showed no obvious metabolic signs of malignant tumors and no obvious abnormal hypermetabolic signs in the brain.

She was diagnosed with KLHL11 encephalitis and was treated with immunoglobulin (0.4 mg/kg for 5 days). In addition, she also received methylprednisolone treatment (500 mg/d, reduced by half every 3 days) and was discharged after 12 days of treatment. After discharge, he was treated with prednisone acetate tablets (40 mg per day, gradually reduced to 8 mg for long-term maintenance) and sodium valproate sustained-release tablets. She was readmitted to the hospital due to recurrent epileptic seizures in September 2023. In addition, her symptoms included cognitive decline, hallucination, restlessness at night and shouting in the sleep. No obvious abnormalities were found in the plain scan and enhanced scan of brain MRI. The electroencephalogram showed that a large number of epileptiform waves could be seen in the bilateral temporal and sphenoid areas during the waking and sleeping stages, especially in the left side of the brain. No abnormalities were found on other test indicators, including hepatitis B virus, cellular immune function (CD3, CD4, CD8, CD16, CD19, CD56), humoral immune function (Ig+C3+C4), and tuberculosis infection T cell detection, and the concentration of valproic acid. Ofatumumab was added to her treatment. She received her first ofatumumab treatment on September 17, 2023. No drug allergies or adverse reactions occurred. After treatment, KLHL11 IgG was detected positive in the serum (1:10).

## Follow-up and outcome

**Figure 3:**
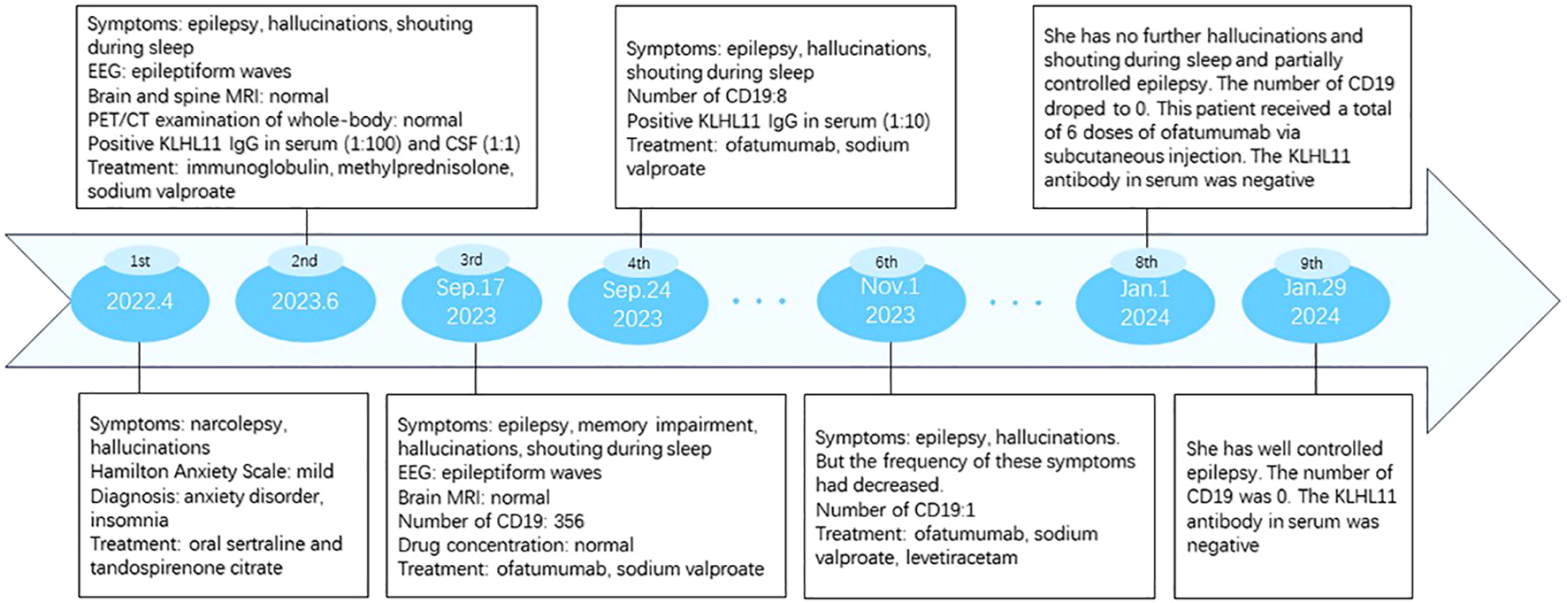
Timeline of disease’s Onset and progression.

**Figure 4:**
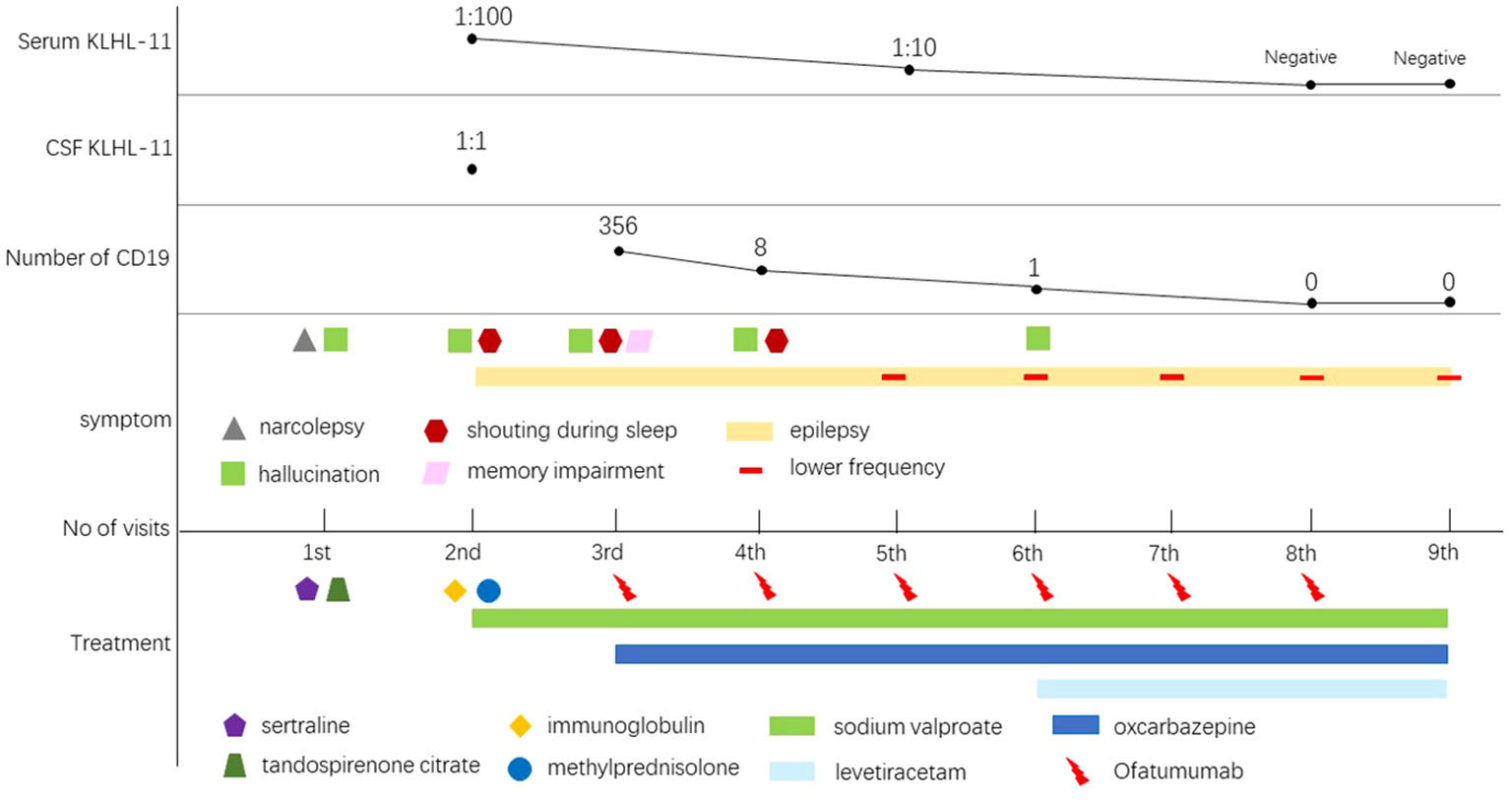
Schematic illustration of the disease course and follow-up. As the patient’s treatment progressed, his antibodies turned negative and his symptoms began gradually to improve.

She received a total of 6 injections of ofatumumab. The number of CD19 decreased from 356 to 0. Her epilepsy symptoms are well controlled. She received levetiracetam in November 2023 to treat epilepsy. The symptoms of hallucinations and yelling during sleep have been greatly improved. No adverse drug reactions occurred. The clinical manifestations, diagnosis, treatment methods and follow-up of the patients are detailed in **Figures 3**, **4**.

## Discussion

This case is a female patient with KLHL11 encephalitis who had a short-term relapse despite immunoglobulin and steroid therapy. Therefore, ofatumumab was used for treatment, and the patient did not relapse after 6 injections. The antibody titer in her serum returned to negative from 1:100, and the number of CD19-labeled cells dropped from 356 to 0. Hallucinations and nighttime yelling have not yet occurred. She had Well-controlled epilepsy.

KLHL11-IgG encephalitis is a rare neurological disease and often causes severe neurological impairment. Currently, the diagnosis and treatment of this disease are only based on case reports or case series. The clinical manifestations of KLHL11 encephalitis are varied. The most common clinical manifestation is rhomboencephalitis, which manifests in the brainstem and cerebellum (3). Specific clinical symptoms of KLHL11 encephalitis include ataxia, vertigo, diplopia, dysarthria, oculomotor disorder, and sensorineural hearing loss (7). However, epilepsy and hallucination was the main symptom in this case. Its pathogenesis is currently unclear, but it may be related to a genetic predisposition to potentially autoimmune tumors and human leukocyte antigens (7).

KLHL11 is a member of the E3 ubiquitin-protein ligase complex involved in protein ubiquitination (8). Studies have reported that in the Olmsted County of Minnesota, the prevalence of KLHL11 encephalitis is estimated to be 2.79 cases per 100,000 males and the incidence rate is 0.21 cases per 100,000 people per year (1). There are more males than females (72/83, 86.7%) among the number of patients with KLHL 11 encephalitis (9). However, there is a lack of reports on the prevalence and incidence of this disease in women. KLHL11 encephalitis is often associated with testicular seminoma. Studies have also reported that this encephalitis may be associated with ovarian teratoma, small cell lung cancer, lung adenocarcinoma, ovarian cancer, chronic lymphocytic leukemia and squamous cell carcinoma (10–12). KLHL11 encephalitis is a rare neurological disease that may be complicated by tumors and severe neurological damage. In addition to supportive care, it is very important to start immunotherapy and screen for tumors as early as possible (14).

Neuropathological studies revealed that the brain of KLHL11 encephalitis patient exhibited chronic lymphocytic inflammation, mainly T cell infiltration, accompanied by non-necrotizing granulomas (3). This study demonstrates that cytotoxic T cell-mediated immune effects play a crucial role in the pathogenesis of KLHL 11 encephalitis (13). Autopsy results revealed that the cerebellum showed moderate to severe loss of Purkinje neurons. Studies suggest that patients with KLHL11 encephalitis have a more difficult-to-treat course than patients with other antibodies against neuronal cell surface antigens (15). Clinicians often use corticosteroids, intravenous immune globulin, or plasma exchange as first-line treatments. In this case, the patient relapsed rapidly after standard treatment with intravenous immunoglobulin and steroids, and received the treatment with ofatumumab. Ofatumumab is a CD20 monoclonal antibody that selectively induces B cell depletion.

After treatment with ofatumumab monotherapy, the patient’s neurological function gradually recovered and the dependence on caregiving decreased. The patient’s symptoms improved significantly after the fourth injection of ofatumumab. This is the first report of ofatumumab in the treatment of KLHL11 encephalitis. Ofatumumab displays specific CD20 affinity and a slow off-rate through complement-dependent cytotoxicity, leading to efficient B cell lysis. Ofatumumab was the first monoclonal anti-CD20 antibody approved for use against human disease and recognizes a different epitope that is distinct from rituximab (16). Ofatumumab was approved for the treatment of relapsing forms of multiple sclerosis in 2020 (17). It can be done by yourself by subcutaneous injection. Ofatumumab is a fully human antibody with unique advantages. It is less immunogenic than rituximab and has a good safety profile (17). The current regimen of ofatumumab for the treatment of multiple sclerosis is 20 mg subcutaneously on days 1, 7, 14, and 28, followed by 20 mg subcutaneously every 4 weeks (18). In this case, we followed the multiple sclerosis treatment protocol. In order to detect efficacy and safety, we measured B cell levels. The B cells in the blood had almost disappeared after the sixth injection of ofatumumab.

Although KLHL11 encephalitis is mediated through T cell immunity, the favorable tried treatment of CD20 monoclonal antibodies in KLHL11 encephalitis may suggest that inducing B cell depletion is an effective and safe option. This mechanism may involve B cell depletion reduces the production of KLHL11 antibodies and inhibits the antibody-associated paraneoplastic neurological syndrome. In addition, B cell depletion inhibits autoreactive T cell responses to restrict the inflammatory cascade and accelerate recovery in KLHL11 encephalitis.

However, more research is urgently needed to explore the clinical efficacy and possible mechanisms of induced B cell depletion in the treatment of KLHL11 encephalitis.

## Conclusion

KLHL11 encephalitis is a very rare disease, especially in women. Current research suggests that cytotoxic T cell-mediated immune responses play an important role in KLHL11 encephalitis. However, in this case, a satisfactory clinical outcome was observed using a CD20 monoclonal antibody that induced B cell depletion in the treatment of KLHL-11 encephalitis. This is the first report of CD20 monoclonal antibody in the treatment of KLHL-11 encephalitis. This case report suggests that depleting B cells may be an effective treatment for KLHL-11 encephalitis. Notably, subcutaneous injection of a CD20 monoclonal antibody that induces B cell depletion may be a viable alternative for the treatment of KLHL-11 encephalitis.

## Data Availability

The original contributions presented in the study are included in the article/supplementary material. Further inquiries can be directed to the corresponding author.
